# Methods of Skull Base Repair Following Endoscopic Endonasal Tumor Resection: A Review

**DOI:** 10.3389/fonc.2020.01614

**Published:** 2020-08-11

**Authors:** Cathal John Hannan, Eoin Kelleher, Mohsen Javadpour

**Affiliations:** ^1^Department of Neurosurgery, Manchester Centre for Clinical Neurosciences, Manchester, United Kingdom; ^2^Nuffield Department of Population Health, University of Oxford, Oxford, United Kingdom; ^3^National Neurosurgical Centre, Beaumont Hospital, Dublin, Ireland; ^4^Royal College of Surgeons in Ireland, Dublin, Ireland; ^5^School of Medicine, Trinity College Dublin, Dublin, Ireland

**Keywords:** skull base, endoscopic, CSF leak, nasoseptal flap, lumbar drain, pituitary, meningioma, chordoma

## Abstract

Following the introduction of fully endoscopic techniques for the resection of pituitary tumors, there was a rapid expansion of the indications for endonasal endoscopic surgery to include extrasellar tumors of the skull base. These techniques offer significant advantages over traditional open surgical approaches to the skull base, including improved tumor resection, and better post-operative neurological outcomes. Following their introduction, however, the initial rate of post-operative CSF leak was unacceptably high. Post-operative CSF leak following skull base surgery is a major source of morbidity, and can lead to the development of life-threatening intracranial infection. The use of vascularized naso-septal flaps transformed the management of these patients, significantly reducing the rate of post-operative CSF leak and increasing the number of patients that could benefit from this less invasive treatment modality. Adequate repair of iatrogenic defects in the skull base is of crucial importance for patients with skull base tumors, as the development of a post-operative CSF leak, and the associated complications can significantly delay the administration of the adjunctive oncological therapies these patients require. In this review, we provide an overview of the latest evidence regarding skull base reconstruction following endoscopic skull base surgery, and describe the skull base repair technique in use at our institution.

## Introduction

The endoscopic endonasal approach to the skull base was initially introduced as an adjunct to the microscope in the resection of pituitary tumors in 1979, with fully endoscopic approaches described in the early 1990s (1–3). The endoscope has since come to supersede the operative microscope in pituitary surgery, due to the improved visualization offered as a result of a wider field of vision, better illumination of the operative field and the ability to inspect anatomical areas using angled endoscopes that are impossible to see using the microscope (4, 5). Following the adoption of this technique for the resection of pituitary tumors, it was adapted for resection of extrasellar skull base lesions (6–9). Fully endoscopic approaches are now in widespread use in the management of malignancies of the ventral skull base, including esthesioneuroblastoma, chordoma, and chondrosarcoma, as well as aggressive, locally invasive pathologies such as meningiomas, and craniopharyngiomas (10–14).

The advantages of these extended endonasal approaches (EEA) to skull base tumors are that they provide a direct trajectory to lesions of the ventral skull base, avoiding the parenchymal retraction and the traversal of cranial nerves inherent to transcranial approaches for these tumors. This less invasive approach is associated with better neurological outcomes and a shorter length of stay than more traditional open approaches (15, 16). When used in the management of chordomas and esthesioneuroblastomas, the endoscopic approaches offer higher rates of gross total resection than their transcranial alternatives (14, 17). However, early series utilizing these approaches were complicated by post-operative CSF leak rates as high as 40%, and this shortcoming was regarded as a major obstacle in their widespread adoption (18). Post-operative CSF leak is the major source of morbidity following endoscopic skull base surgery, and can lead to the development of meningitis and/or hydrocephalus (19, 20). Moreover, CSF leak leads to longer length of stay and increases the chances of unplanned readmission to hospital following surgery, both of which have the potential to delay, or interrupt adjunctive therapy in patients with skull base malignancies (21, 22).

The resection of pituitary adenomas is often an extra-arachnoidal procedure, with a small dural defect created to access the pathology and is therefore associated with a low rate of CSF leak that was not observed to increase following the introduction of the endoscopic technique (23). EEA to skull base malignancies, however, necessitate larger bony and dural defects, causing high flow intra-operative CSF leaks which are demonstrably associated with higher rates of post-operative CSF leak (24). Therefore, the reconstruction of the skull base following extended EEA to the skull base is of paramount importance in avoiding post-operative CSF leak. Advances in skull base reconstruction, particularly the use of vascularized local flaps, have greatly reduced the incidence of this complication and have been instrumental in the expansion of these approaches for the management of skull base malignancy (25). The importance of using vascularized flaps as part of a multi-layer, rather than a monolayer closure of skull base defects to prevent post-operative CSF leak has also been highlighted in a recent study by Simal-Julián et al. (26). In this review, we will provide an overview of the latest methods used to reconstruct large skull base defects leading to high flow CSF leaks following tumor resection, as well as describing our preferred method for the repair of these defects.

## Discussion

### Skull Base Reconstruction Methods

#### Pedicled Nasoseptal Flap

The development of the naso-septal flap (NSF) in by Hadad et al. in 2006 revolutionized the field of endoscopic endonasal skull base surgery and has facilitated the expansion of this treatment modality (27). Prior to its development, skull base repair was undertaken using multilayered techniques employing autologous fat grafts and synthetic dural substitutes as inlay and onlay grafts secured with fibrin glue, which could be supported by the intranasal placement of Foley catheters (28). As mentioned above, this repair technique was associated with an unacceptably high rate of post-operative CSF leak and the requirement for an alternative technique was clear. The NSF consists of a vascularized mucoperichondrial/periosteal flap harvested from the midline nasal septum and pedicled on the posterior septal branch of the sphenopalatine artery. This allows for the creation of a large flap, capable of covering skull base defects extending from the frontal sinus to the sella antero-posteriorly, and spanning the width of the distance between both orbits. This vascularized flap was used in combination with an inlay synthetic collagen graft and an autologous fat graft, secured using fibrin glue. In a series of 44 patients undergoing endoscopic skull base surgery involving large dural defects and high flow intra-operative CSF leaks, the authors reported a post-operative CSF leak rate of 4.5% (27). In the setting of very large skull base defects, involving the anterior and posterior cranial fossa, bilateral NSF have been harvested to effectively prevent CSF leak (29).

This technique was widely adopted soon after its introduction, and Kassam et al. published their experience of NSF utilization in 75 patients following EEA to a variety of skull base tumors. A large dural defect with high flow intra-operative CSF leak was noted in 55 patients. In similar fashion to that reported by Hadad et al. the authors combined the nasoseptal flap with the use of an inlay synthetic dural graft, secured using a biological glue or Foley catheter. In the first 1/3 of the series, the authors noted a post-operative CSF leak rate of 33% in cases with a high-flow intra-operative CSF leak rate, which dropped to 5.4% in the latter 2/3 of the series (30). With craniopharyngiomas in particular, the authors noted in a separate publication that the use of a NSF dramatically decreased the rate of post-operative CSF leak from 58 to 5% (31). As a testament to the versatility of this technique, it has also been successfully utilized following EEA to skull base lesions in pediatric cohorts, in spite of initial concerns regarding the small size of the nasal septum in children (32, 33). Certain skull base tumors, such as craniopharyngiomas, chordomas and chondrosarcomas have a propensity for local recurrence, necessitating revisional surgery for further tumor resection. NSF can be successfully re-used in this setting, by dissecting it from the initial defect site and re-applying it in the standard fashion, with no increase in the rate of post-operative CSF leak (34). Traditional open approaches to skull base tumors are often closed with local vascularized pericranial flaps, and the options for skull base defect repair in the setting of a post-operative CSF leak can be limited. The use of an endoscopically harvested NSF to successfully control CSF leak following open skull base surgery has been reported, expanding the repertoire of this technique even further (35).

Although the development of the NSF was a significant advance in skull base surgery, the technique itself is subject to some limitations. Although it is a rare occurrence, the flaps are subject to necrosis due to compromise of the vascularized pedicle: this is reported to occur in <1% of cases, but these patients will often require revisional surgery for alternative skull base reconstruction (36, 37). The removal of the mucosa from the nasal septum leaves a large defect, that heals by secondary intention over an extended period. This process can lead to significant nasal crusting and a perception of nasal obstruction in the ipsilateral nostril (38). More significant structural abnormalities of the nose can also occur, such as septal perforations and collapse of the nasal dorsum, with the rates of these complications varying from 1 to 14% in the published literature (37, 39, 40). Overall, the use of a NSF for skull base reconstruction can lead to additional morbidity due to the sinonasal complications associated with this technique. A recent review of over 700 patients who underwent endoscopic skull base surgery found that the use of a NSF conferred additional sino-nasal morbidity post-operatively, and had a negative impact on the sino-nasal quality of life outcomes of patients (41).

The NSF is an effective, versatile technique that has gone on to form the basis of skull base reconstruction protocols in a number of high-volume skull base centers the world over, with some modifications which will be explored in the sections that follow. Table 1 summarizes the results of the use of the NSF within skull base reconstruction protocols following EEA and resection of skull base tumors.

**TABLE 1:** Table summarizing the results of studies using a vascularized nasoseptal flap following EEA and intra-operative CSF leak.

Author, Year	Technique	Number of Cases	Post-operative leaks (%)
Hadad et al., 2006 (27)	Collagen inlay graft ± fat graft + NSF + fibrin glue + nasal packing/Foley catheter	44	2 (4.5)
Kassam et al., 2008 (30)	Collagen inlay graft ± fat graft + NSF + dural sealant + nasal packing/Foley catheter	55	8 (14.5)
Zanation et al., 2009 (71)	Collagen inlay graft + NSF ± fat graft + DuraSeal^®^ + Gelfoam^®^ nasal packing/Foley catheter	70	4 (5.7)
Luginbuhl et al., 2010 (43)	Dual layer “button” fascia lata graft + NSF + dural sealant + Nasopore^®^	16	1 (6.3)
Liu et al., 2012 (61)	Fascia lata graft inlay/overlay graft ± fat graft + Surgicel^®^ ± fascia lata + NSF + Merocel^®^ tampon	93	3 (3.2)
Garcia-Navarro et al., 2013 (42)	Fat graft + onlay fascia lata + MEDPOR/Bone + NSF + DuraSeal^®^ ± Lumbar Drain	21	1 (4.7)
Thorp et al., 2014 (37)	NSF ± middle turbinate graft (no further details provided)	144	3 (2.1)
Cavallo et al., 2019 (44)	Fat graft + NSF + Merocel^®^ tampon	25	1 (4)
Conger et al., 2019 (67)	Fat graft + collagen sponge + MEDPOR/Bone + NSF + Fat graft + collagen sponge + dural sealant + Merocel^®^ tampon	83	4 (4.3)

#### Gasket Seal Technique

Long-term outcomes from a series of 46 patients who underwent EEA and skull base reconstruction using the gasket seal technique were published by Garcia-Navarro et al. in 2013 (42). This technique involves the placement of an autologous fat graft to eliminate intracranial dead space, covered by an autologous fascia lata graft over the bony skull base defect, with the fascial graft sized such that it extends 1 cm beyond the defect circumferentially. Following the placement of this graft, an autologous bone graft, or synthetic polyethylene implant is laid over the fascial graft, sized to fit snugly inside the bony defect. In the latter stages of their series, the authors then placed a NSF over this solid buttress, secured with DuraSeal (Confluent Surgical, United States). In 67% of cases, this repair technique was combined with a 24–48 h period of prophylactic lumbar drainage. In the cases where the gasket seal technique was combined with a NSF, the authors reported a post-operative CSF leak rate of 4.7%. The authors commented that as the solid buttress they use is not curved, this technique is suboptimal for the closure of large skull base defects that cross two geometric planes (e.g., anterior skull base and clivus).

#### Bilayer Button Technique

This technique, originally described by Luginbuhl et al. in 2010 utilizes a bilayer fascia lata graft, in conjunction with a NSF. In this method, the authors suture an onlay fascia lata graft slightly larger than the bony defect onto a much larger piece of fascia lata that goes on to act as an inlay graft. The inlay graft is directly opposed to the dura, with the appropriately sized onlay graft acting to prevent graft migration from the dural defect. This fascial construct was then covered in 16/20 cases by a NSF, secured with a fibrin glue. Using this technique, the authors noted a decrease in the rate of post-operative CSF leak in patients with large dural defects from 45 to 10% (43). Although the authors introduced the sutured fascia lata construct at the same time as the NSF, given the results from other series, it is highly likely the greatest contributor to the decreased rate of post-operative CSF leak was the NSF.

#### The 3F Technique

Cavallo et al. recently published a modification to their skull base reconstruction technique following EEA, having previously employed a modification of the gasket seal technique combined with a NSF (10). In this modification, which the authors call the 3F technique, the first F (fat) is the placement of an autologous fat graft into the dead space created by tumor resection, which acts to span the entirety of the osteodural defect, secured with fibrin glue. The second F (flap) is the placement of the NSF, bolstered with cellulose sponges, and secured with nasal tamponades for 72 h. The authors mobilize the patient to a sitting position as soon as possible after surgery and they are encouraged to walk and stand as much as possible, the third F (flash). Using this skull base reconstruction protocol, the authors reported a post-operative CSF leak rate of 4% in 25 patients who had large osteodural defects following EEA (44). Post-operative lumbar drainage was not used.

### Alternative Options

In situations where the pedicled NSF is not available, for example when sinonasal malignancies invade the nasal septum or pterygopalatine fossa, or when the patient has undergone previous reconstruction with a NSF, alternative vascularized regional flaps are available. The lateral nasal wall flap is harvested from the opposite side of the nasal cavity to the NSF, and is based on the lateral nasal wall artery, a branch of the sphenopalatine artery. In a series of 24 patients with high flow intra-operative CSF leaks, Lavigne et al. reported a post-operative CSF leak rate of 25% (45). Although at first glance this figure appears to be high, it should be borne in mind that this reconstructive method was used as a salvage method, after necrosis of an existing NSF or when the a NSF was not available, having been used in previous surgery. The authors comment that the lateral nasal wall flap cannot cover as great a surface area as the NSF, and due to the fact it is harvested from the conchal surfaces of the lateral nose, it has a greater “memory” and may migrate from its intended position more often. When local vascularized reconstruction options are not available, due to extensive tumor invasion/previous radiotherapy, or where the expertise in vascularized flap reconstruction does not exist, avascular free grafts are an option. In this technique, layered reconstruction of the skull base defect created following EEA is undertaken using a variety of autologous and non-autologous dural substitutes. In a large series of EEA to skull base tumors, Roxbury et al. reported a post-operative CSF leak rate of 6.85%, using a multi-layer closure consisting of an underlay layer of synthetic dural substitute or fat graft, an overlay layer of dural substitute and a further overlay layer of Alloderm^®^ (Lifecell, United States) acellular matrix in combination with a free mucosal flap (46). However, the authors noted that on multi-variate analysis that a high-flow CSF leak, as is often generated in EEA to skull base tumors, was associated with a higher rate of post-operative CSF leak and the majority of the cases in their series were pituitary adenomas, which are known to be associated with a lower rate of post-operative CSF leak (31). More convincing evidence of the potential efficacy of free graft reconstruction techniques is provided by a recent study published by Matavelli et al., wherein the authors describe the results following the resection of 186 sinonasal malignancies, resulting in large anterior cranial fossa defects. Using autologous iliotibial tract and fat tissue in a three-layer reconstruction protocol, the authors reported a post-operative CSF leak rate of 5.8% (47). Although these studies do suggest that acceptable results can be obtained with the use of free graft techniques, in the absence of a trial comparing both techniques, the weight of the evidence suggests that lower rates of CSF leak are obtained with the use of local vascularized flaps, and this view is supported by the results of a systematic review comparing the efficacy of skull base reconstruction methods following EEA (48). A further reconstruction option in the context of unavailability or unsuitability of the NSF is the endoscopic pericranial flap. This technique, originally described by Zanation et al., involves the minimally invasive, endoscopic harvesting of a pericranial flap through a small scalp incison. This flap is then brought through into the nasal cavity via a bony defect drilled in the nasion (49). Following this, it can be used to cover osteodural defects in an identical manner to the NSF and it has been successfully utilized in the reconstruction of anterior and posterior fossa skull base defects (50, 51).

In the setting of previous radiotherapy to the skull base, resulting in delayed CSF leak, transposition of a temporo-parietal fascial flap pedicled on the superficial temporal artery has been utilized (52, 53). This involves harvesting of the flap through an external skin incision overlying the temporal fossa, which is then transposed through the infratemporal fossa into the nasal cavity via an endoscopic *trans*-maxillary sinus or *trans*-pterygoid approach. Although there are reports of its success, the requirement for an external skin incision, as well as the risk of injury to the frontal branch of the facial nerve mean this approach is uncommonly used, and reserved for when local flap options are unsuitable.

In the setting of locoregional flap failure, the use of free myo-cutaneous flaps, facilitated by microvessel anastomosis to reconstruct skull base defects following EEA has been described. Kang et al. have described the successful use of a vastus lateralis flap, pedicled on the descending branch of the lateral femoral circumflex artery in four patients with anterior skull base defects following EEA. In all four cases, initial locoregional flap methods failed to adequately reconstruct the skull base and the vastus lateralis flap was employed as a salvage procedure, whereby the facial artery was used as a recipient vessel and the flap was tunneled through a maxillotomy to cover the skull base defect (54). These techniques have also been utilized in the repair of posterior fossa defects following EEA; the radial forearm free flap has been employed effectively to reconstruct a cranio-cervical junction defect following EEA for a clival chordoma. Similar to the four cases above, the patient had undergone previous attempts to reconstruct the skull base using a NSF (55). The use of free flaps for the reconstruction of the skull base following EEA is a significant undertaking, requiring complex mutli-discplinary input and in our view should only be considered when loco-regional reconstruction methods have failed.

### Adjuncts to Skull Base Repair

#### Lumbar Drainage

The value of post-operative lumbar drainage of CSF following EEA to the skull base has been the source of debate since the introduction and widespread adoption of these approaches. The initial high rate of post-operative CSF leak with EEA prompted some centers to adopt lumbar drainage as a matter of course following EEA, providing observational evidence for their efficacy (56). Others called into question the necessity of lumbar drainage when a NSF is used, and suggested they may in fact be harmful, citing the risk of meningitis, CSF over-drainage and spinal headache and longer hospital stay with their use (57–59). In reality, the heterogeneity of the skull base repair methods in these studies, as well as their observational nature leaving them highly susceptible to selection bias, limited the conclusions that could be drawn from them.

The requirement for a randomized controlled trial, with clearly defined inclusion criteria and controls in place for selection bias was clear, and the results from such a trial were published in 2018. In this trial, published by Zwagerman et al. all patients undergoing EEA resulting in a dural defect >1 cm^2^ along with extensive arachnoid dissection and/or entry into a ventricle were eligible for recruitment (60). Patients were randomized to drain or no drain after the completion of skull base reconstruction, with the lumbar drain placed in the operating room and left in place for 72 h, draining 10 ml/h. All patients had skull base reconstruction with a local vascularized flap. The trial was terminated early having recruited 170 patients, due to evidence of benefit in the lumbar drain arm of the trial. 18/85 (21.2%) of patients with no drain suffered a post-operative CSF leak compared with 7/85 (8%) of patients who had a lumbar drain placed. There were no instances of meningitis associated with lumbar drain use, and only two patients developed spinal headache requiring a blood patch. There was also no significant increase in the risk of venous thromboembolism in the patients who had a lumbar drain placed. In a subgroup analysis based on lesion location, the authors concluded that there was a significant decrease in the incidence of post-operative CSF leak with use of a lumbar drain in patients with pathology located in the anterior and posterior cranial fossa, but that patients with tumors in the suprasellar area did not benefit from lumbar drain insertion. The authors suggested this may have been because the vascularized local flaps used are most effective in the suprasellar region, but they may not provide enough coverage to cover larger defects anteriorly and posteriorly. The results from this trial are striking, but should be interpreted with caution given that this a single center study where one skull base reconstruction protocol is used; the applicability of these results to centers utilizing different methods of skull base repair are uncertain. Moreover, the rates of post-operative CSF leak in both groups were higher than those previously reported in defects closed with vascularized local flaps, and the authors did not provide any data on length of stay in the two cohorts (27, 30, 42). Despite the shortcomings of this trial, it is likely that there are a subset of patients at particularly high risk of CSF leak that stand to benefit from “prophylactic” lumbar drain insertion.

#### Direct Support of Repair

Following the positioning of the materials used in the skull base reconstruction, the majority of authors would advocate some form of physical support for the reconstruction, to allow time for epithelisation of the defect and for the mucosa of the NSF to integrate with the mucosa adjacent to the operative site. A number of series have utilized the placement of a Foley catheter with the balloon inflated to provide an upward pressure on the skull base repair, whereas others use nasal tampons or inflatable Merocel^®^ (Medtronic, United States) sponges (30, 61, 62). Prior to the insertion of any buttressing material, the use of tissue sealants to secure the NSF to the skull base is commonplace, although Liu et al. argue that this is not required and merely contributes to unnecessary surgical costs (30, 42, 62, 63).

A further technique to provide support for skull base reconstructions following EEA that has been suggested is the suturing of an onlay fascia lata graft to the edges of the dural defect, following the placement of an inlay synthetic dural substitute in the subdural space and combined with a NSF. Xue et al. found that the rates of post-operative CSF leak decreased following their implementation of this practice, although this was confounded by the fact that there was a significantly higher rate of intra-operative lumbar drain insertion in the group with dural suturing (64). The requirement for dural suturing has also been reported in endoscopic re-intervention for post-operative CSF leak, but at present there is no evidence to support its routine use in all EEA for skull base tumors or for its superiority over non-suture techniques (42, 65).

### Skull Base Repair: The Dublin Technique

In our center, we employ a standardized method of skull base reconstruction for all EEA as well as endoscopic *trans*-sphenoidal approaches to pituitary tumors, even in the absence of an intra-operative CSF leak. Following the establishment of this protocol, we have reported a post-operative CSF leak rate of 1%, although this was higher in the early part of the senior author’s experience prior to the introduction of this standardized technique, in keeping with the experience of other surgeons (30). In the latter third of a series of 270 patients (operated between July 2006 and June 2016) undergoing endoscopic surgery for resection of pituitary and skull base tumors, 1/90 (1%) patients experienced a post-operative CSF leak. When only EEA with high flow intra-operative CSF leaks were repaired using the following technique, 1/28 (4%) of patients experienced a post-operative CSF leak (66).

**FIGURE 1:**
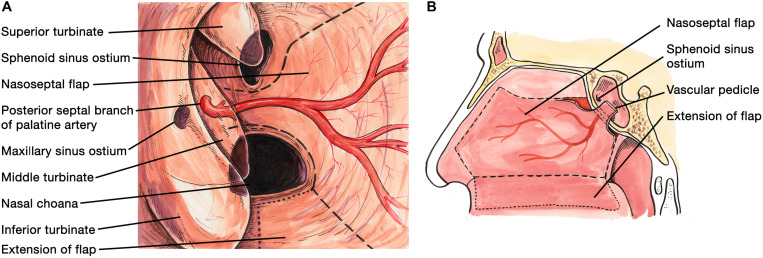
**(A)** Diagrammatic representation of the endoscopic view during harvesting of the naso-septal flap via the right nostril. An incision is made 1–2 cm below the cribriform plate along the mucosa of the nasal septum. A further incision is made along the medial aspect of the floor of the nasal cavity, which can be extended further medially (dotted line) if a large naso-septal flap is required. Both incisions are then connected by an anterior vertical incision. The flap is then dissected from the nasal septum in retrograde fashion and stored in the nasopharynx or maxillary sinus, to be used for skull base reconstruction at the end of the case. **(B)** Sagittal view of the boundaries of the nasoseptal flap, with the dotted line indicating an optional extension of the incision if a large flap is required.

**FIGURE 2:**
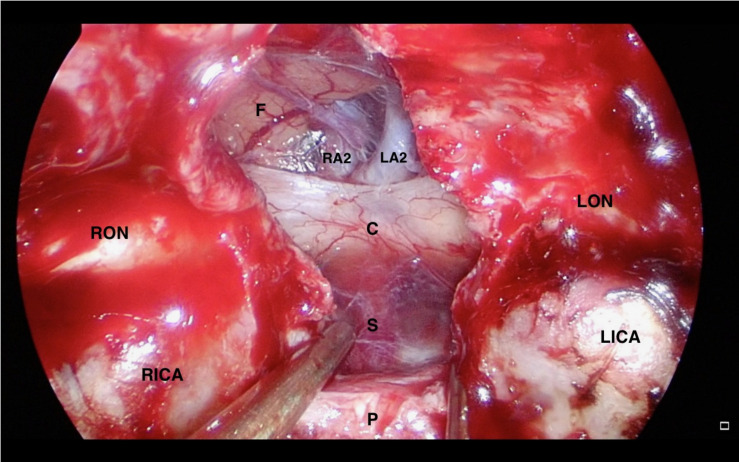
Intra-operative endoscopic view of the skull base defect following a *trans*-tubercular approach to a planum sphenoidale meningioma. C: Optic Chiasm, F: Frontal Lobe, LA2: A2 segment of left anterior cerebral artery, LICA: Left Internal Carotid Artery, LON: Left Optic Nerve, P: Pituitary Gland, RA2: A2 segment of right anterior cerebral artery, RICA: Right Internal Carotid Artery, and RON: Right Optic Nerve.

**FIGURE 3:**
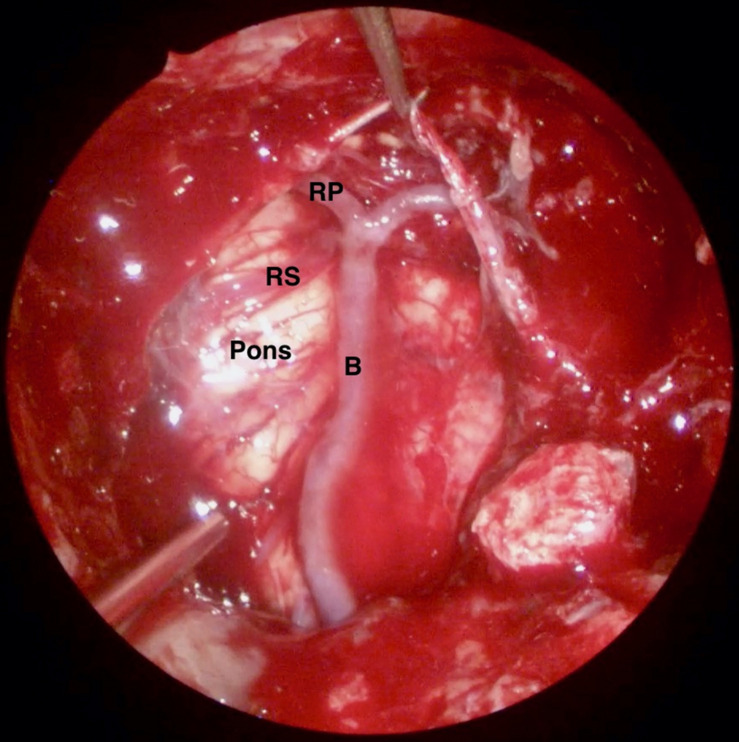
Intra-operative endoscopic view of the skull base defect following the resection of a clival chordoma. B: Basilar Artery, RP: P1 segment of right posterior cerebral artery, and RS: Right superior cerebellar artery.

**FIGURE 4:**
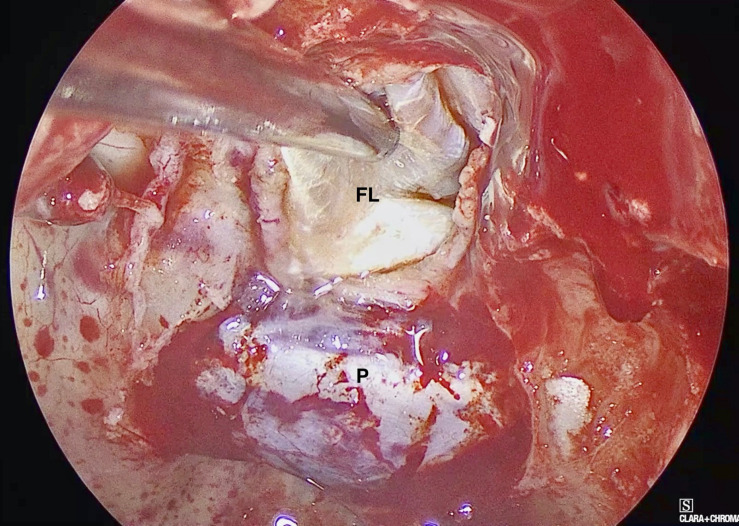
Intra-operative endoscopic view demonstrating the placement of the inlay fascia lata graft. FL: Fascia Lata, P: Pituitary Fossa Dura.

**FIGURE 5:**
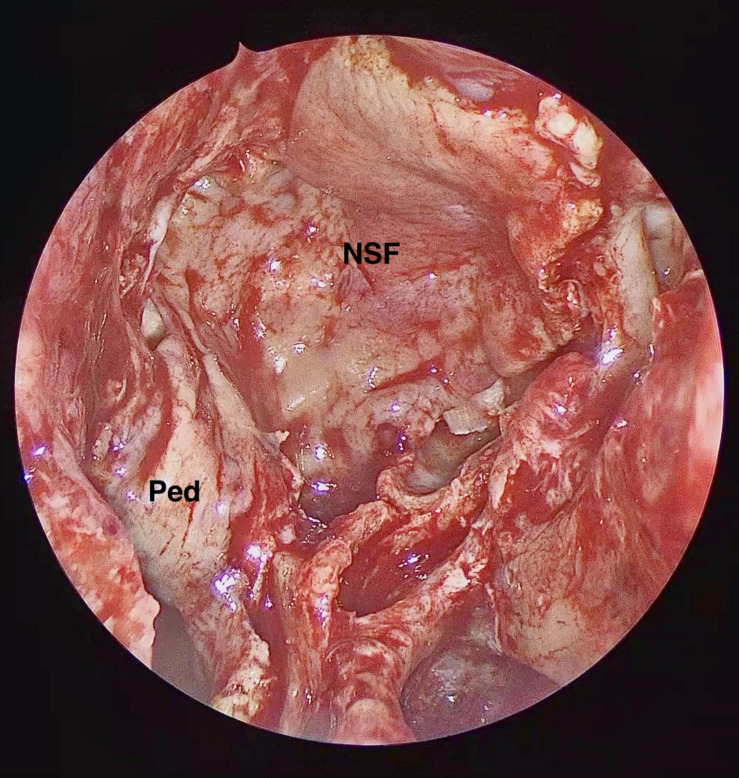
Intra-operative endoscopic view demonstrating the naso-septal flap placed to cover the osteo-dural defect in its entirety. NSF: Nasoseptal flap, Ped: Vascular Pedicle.

**FIGURE 6:**
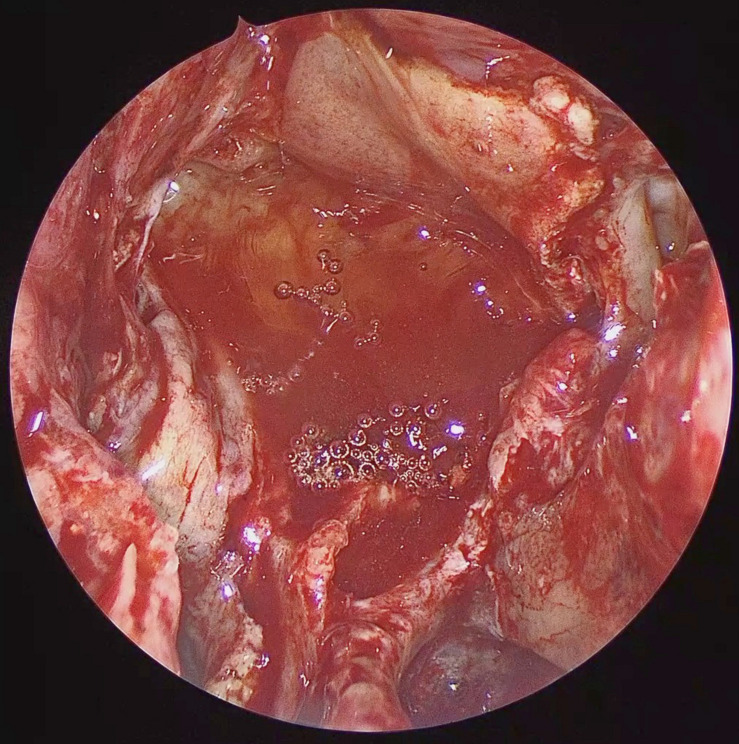
Intra-operative endoscopic view of the naso-septal flap secured with dural sealant.

A NSF flap is harvested at the beginning of the procedure, and stored in the posterior nasopharynx/maxillary sinus until completion of the tumor resection. Figure 1 is a diagrammatic representation of the harvesting of a NSF at the beginning of the procedure. Following tumor removal, an inlay graft of autologous fascia lata is inserted in the subdural space, and is sized to be larger than the osteodural defect in all dimensions. The only fascia lata donor site complication in our series was one case of wound hematoma requiring evacuation in a patient with Cushings disease (1/28, 4%). Figures 2, 3 are intra-operative photographs demonstrating the variety of skull base defects that can be closed using this technique. Placement of the fasica lata as an inlay larger than the dural opening ensures that the graft does not migrate out of the defect, and that it is opposed to the dura mater with each CSF pulsation. This intradural fascial layer is not secured using sutures/clips and contrary to concerns raised by some authors, we have not noted any issues regarding migration of the graft material (43). We then place the vascularized NSF directly over the dural and bony defects, with no intervening exogenous material. We avoid placing any intervening material between the dura and the NSF because in our view, natural tissues with good blood supply are more likely to adhere to each other and any intervening material may hinder this. The NSF is then covered with a further layer of fascia lata, and the entire construct is secured with dural sealant. Our preferred dural sealant is Bioglue^®^ (CryoLife, Inc., United States). Figures 4–6 demonstrate the major components of our skull base repair technique. We do not insert further bolstering materials (Foley catheters, nasal tampons) and we do not use any prophylactic lumbar drains. Prior to the adoption of this technique in 2013, we utilized a fat graft, covered with an onlay graft of dural substitute/fascia lata secured with dural sealant. In the setting of a high flow intra-operative CSF leak, a post-operative CSF leak was noted in 7 of 20 cases (35%) (66).

**FIGURE 7:**
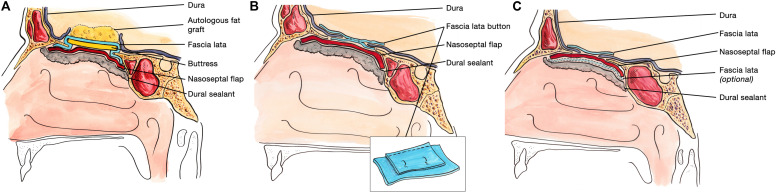
Comparative sagittal view of the Gasket seal technique **(A)**, the Bilayer button technique **(B)**, and the Dublin technique **(C)** for skull base reconstruction following endoscopic endonasal resection of skull base tumors. **(A)** The Gasket seal closure technique consists of the placement of an autologous fat graft to eliminate dead space (this is not used when the 3rd ventricle has been opened), with a layer of fascia lata larger than the dural defect wedged in place with a solid buttress of bone or MEDPOR. This construct is then covered with a nasoseptal flap and secured with DuraSeal^®^. **(B)** In the bilayer button technique, two pieces of fascia lata are sutured together, with one much larger than the other. The larger piece of fascia lata is placed intradurally, and the smaller piece placed on the outside of the dural defect. This is then covered with a nasoseptal flap and secured with NasoPore and dural sealant. **(C)** In the Dublin technique, a fascia lata graft larger than the dural defect is placed intradurally as an inlay graft. This fascia lata graft is covered directly by a NSF, which may be secured with a further layer of fascia lata. Bioglue^®^ is used to complete the skull base repair. Panel **(A)** adapted with permission from figure in Garcia-Navarro et al. (42). Panel **(B)** adapted with permission from figure in Luginbuhl et al. (43).

Our technique is different from the Gasket-seal technique, insofar as an inlay rather than an onlay fascia lata graft is used, and is more similar to the bilayer button technique and indeed that originally described by Hadad et al. in that respect (27, 42, 43). Figure 7 allows for a comparison of the two major alternatives to our technique. We also diverge from the protocol of Conger et al. who argue that a solid buttress is required for the closure of high flow intra-operative CSF leaks (67). The other published series that most closely resembles our method is that of Eloy et al., describe the use of a NSF to cover an initial layer of autologous fat, fascia lata or dural substitute, secured with dural sealant, and a Merocel tampon. In concordance with our preferred method, the authors do not routinely use a lumbar drain and they reported a post-operative CSF leak rate of 0% in 59 patients with a high flow intraoperative CSF leak.

### Post-operative CSF Leak: Risk Factors

Identification of patients at higher risk of post-operative CSF leak following EEA allows the surgeon to ensure particularly meticulous skull base reconstruction following tumor resection, as well as considering the pre-emptive insertion of a lumbar drain. A number of studies have been performed to identify these risk factors, and BMI ≥ 30 has frequently been identified as being associated with an increased risk of post-operative CSF leak (31, 68, 69). The presence of an intra-operative CSF leak is strongly associated with a greater risk of post-operative CSF leak, as highlighted by the much higher rates of this complication in patients undergoing EEA compared to those having endoscopic *trans*-sphenoidal approaches to pituitary tumors (31).

There is also evidence to suggest that posterior fossa defects have a higher proclivity for post-operative CSF leak, which may not be surprising given their dependent location and the requirement for any vascularized nasoseptal flap to be transposed to a greater extent than if they were being used for an anterior fossa or sellar defect (69, 70). The rate of post-operative CSF leak has been shown to decrease as the experience of the operating surgeon increases, with data from our series of 270 endoscopic cases identifying a CSF leak rate of 21% in the first 90 cases, as compared to 1% in the last 90 cases (66).

## Conclusion

Effective closure of the large osteodural defects created by EEA to skull base tumors is of vital importance in the prevention of post-operative CSF leak and meningitis. The addition of the NSF to multi-layered closure has been transformative in this regard, and as demonstrated in Table 1, has brought the risk of post-operative CSF leak below 5%. The role of routine, pre-emptive lumbar drain insertion requires further clarification but one randomized controlled trial has shown benefit in selected cases.

## Author Contributions

CH and MJ drafted and reviewed the manuscript. EK created the medical illustrations. All authors contributed to the article and approved the submitted version.

## Conflict of Interest

The authors declare that the research was conducted in the absence of any commercial or financial relationships that could be construed as a potential conflict of interest.
